# Supplementary material for: Trifloxystrobin blocks the growth of Theileria parasites and is a promising drug to treat Buparvaquone resistance
Source: Commun Biol. 2022 Nov 15;5:1253. doi: 10.1038/s42003-022-03981-x (PMC9666453; doi:10.1038/s42003-022-03981-x)
Supplement: Supplementary file 2 — Supplementary information [file 42003_2022_3981_MOESM2_ESM.pdf]

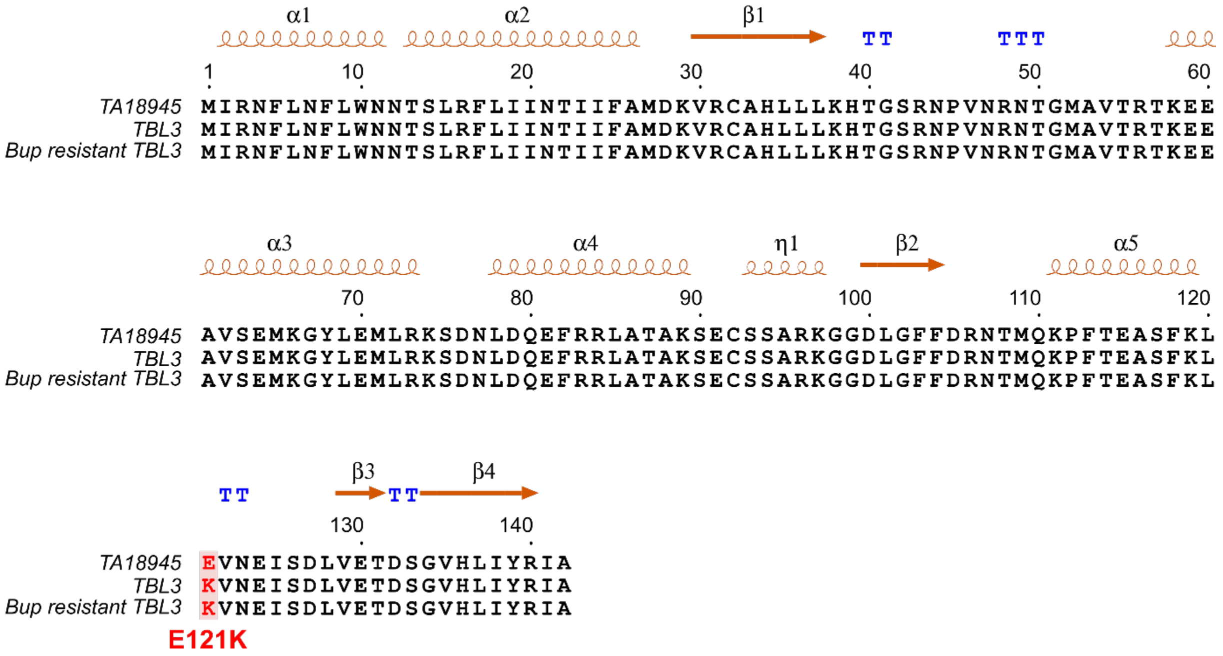


**Supplementary Figure 1: Sequence of the *TaPin1* gene in TBL3 cell lines**

Sequence alignment of TaPin1 proteins in TBL3 parental cells and Buparvaquone-resistant TBL3 cell lines. The sequences were identical before and after the generation of drug resistance and both proteins vary slightly from the reference sequence for TaPin1 protein (TA18945 from Piroplasmadb.org).

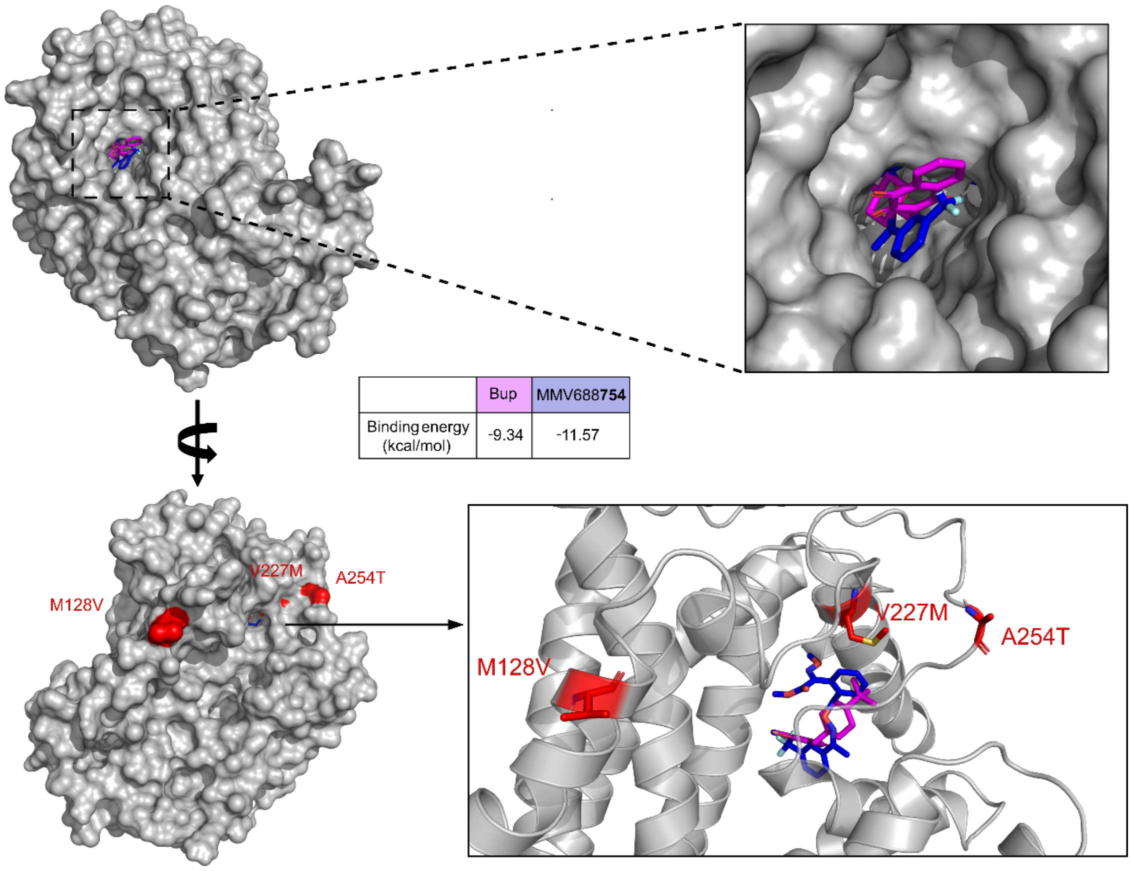


| | Bup | MMV688754 |
|---|---|---|
| Binding energy (kcal/mol) | -9.34 | -11.57 |

**Supplementary Figure 2: Model prediction of TaCytB protein structure and drug interactions**

Homology prediction model for parasite TaCytB protein (in gray) based on high similarity with chicken cytochrome B (4U3F)[38]. Buparvaquone (in purple) and MMV688754 (in blue) were computationally docked in the q0 site of the enzyme and the predicted binding energy of each compound-enzyme complex was evaluated. TaCytB mutations that are present in the Buparvaquone-resistant TBL3 cell line are displayed in red.

| Compound (Plate_Well) | Survival % of DMSO < 25% | | MMV | Compound name | Family |
|---|---|---|---|---|---|
| | 10 µM | 2 µM | | | |
| A_A11 | 3.6 | | | | |
| A_B11 | 3.2 | 18.4 | MMV676477 | CHEMBL2098184 | TUBERCULOSIS |
| A_C04 | 3.1 | | | | |
| A_F04 | 2.9 | | | | |
| A_H09 | 6.3 | | | | |
| A_H11 | 5.4 | | | | |
| B_A11 | 9.8 | | | | |
| B_B03 | 10.8 | 17.7 | MMV676604 | AZD5438 | KINETOPLASTIDS |
| B_C03 | 21.8 | 6.8 | MMV676600 | Danusertib | KINETOPLASTIDS |
| B_C11 | 4.27 | | | | |
| B_G06 | 4.9 | 0.4 | MMV003152 | Mebendazole | REFERENCE COMPOUNDS |
| B_G10 | 23.4 | | | | |
| B_H02 | 0.02 | | | | |
| B_H03 | 13.8 | | | | |
| B_H08 | 0.05 | | | | |
| C_A05 | 3.2 | | | | |
| C_A10 | 6.8 | | | | |
| C_C03 | 0.9 | | | | |
| C_D11 | 23.5 | | | | |
| C_E11 | 6.7 | | | | |
| C_F04 | 2.9 | | | | |
| C_F08 | 0.8 | | | | |
| C_F10 | 10 | | | | |
| C_G03 | 22.5 | | | | |
| C_G04 | 0.5 | | | | |
| C_G05 | 0.4 | | | | |
| D_A05 | 2.3 | | | | |
| D_B07 | 0.2 | | | | |
| D_B09 | 9.5 | | | | |
| D_B10 | 0.2 | | | | |
| D_D11 | 0.2 | | | | |
| D_E02 | 19.6 | | | | |
| D_E05 | 2.4 | | | | |
| D_F03 | 5.9 | | | | |
| D_F06 | 3.5 | | | | |
| D_F11 | 0.05 | | | | |
| D_G08 | 0.1 | 11.1 | MMV688372 | CHEMBL3115821 | KINETOPLASTIDS |
| D_G11 | 8.9 | | | | |
| D_H09 | 1.1 | 0.9 | MMV675968 | CHEMBL88430 | CRYPTOSPORIDIOSIS |
| E_D05 | 8.2 | | | | |
| E_E11 | 0.6 | 3.7 | MMV153413 | Thiophene-4 | TUBERCULOSIS |
| E_F08 | 0.05 | | | | |
| E_G04 | 4.3 | 2.8 | MMV021013 | CHEMBL530275 | TUBERCULOSIS |
| E_G06 | 9 | 15.9 | MMV688754 | Trifloxystrobin | KINETOPLASTIDS |

## Supplementary Table 1

| Target genes | Primers sequence Forward | Primers sequence Reverse |
|---|---|---|
| Actin | GGCATCCTGACCCTCAAGTA | CACACGGAGCTCGTTGTAGA |
| Tasp | AGCAGCCCCTTGTCATGGG | TAATAGCTTTTGCACGGAGGA |
| MMP9 | CCCATTAGCACGCACGACAT | CACGTAGCCCACATAGTCCA |
| HK2 | CGACCAAGTGCAGAAGGTTG | CGTCTGGAGTAGACCTCAC |
| miR155 | TGGCGCAAACCAGGAAGGGGA | ACAGCCCACAGCAAGCCTCC |
| PDK1 | CTAGGCGTCTGTGTGATTTG | GATAGAGGTGGGATGGTAC |
| HSP70 | ACGCAAATGGAATCCTCAAC | TATTCGTCGTGCTCTGCTAA |
| TamR1 | CCACTCCTGTAGCGGGTAAA | TTGGGAGGTACTGACCCAAA |
| TaCytB | TACCGAGCTCGGATCATGAATTTGTTTAACTCACATTTGC | GCCCTCTAGACTCGATTATGCACGAACTCTTGC |

**Supplementary Table 2**
