# Supplementary material for: Trifloxystrobin blocks the growth of Theileria parasites and is a promising drug to treat Buparvaquone resistance
Source: Commun Biol. 2022 Nov 15;5:1253. doi: 10.1038/s42003-022-03981-x (PMC9666453; doi:10.1038/s42003-022-03981-x)
Supplement: Supplementary file 3 — Description of Additional Supplementary Files [file 42003_2022_3981_MOESM3_ESM.pdf]

Description of Additional Supplementary Files

File name: Supplementary Data 1

Description: The source data behind the graphs and plots in the paper
